# A RPA-CRISPR/Cas12a-Powered Catalytic Hairpin Assembly Fluorescence Biosensor for Duck Plague Virus Virulent Strain Detection

**DOI:** 10.3390/bios16020073

**Published:** 2026-01-26

**Authors:** Yue Wu, Jiaxin Wan, Xingbo Wang, Yunjie Shen, Xiangjun Li, Weidong Zhou, Yinchu Zhu, Xing Xu

**Affiliations:** 1 State Key Laboratory for Quality and Safety of Agro-Products, Institute of Animal Husbandry and Veterinary Science, Zhejiang Academy of Agricultural Sciences, Hangzhou 310021, China; wuyue@zaas.ac.cn (Y.W.); wangxingnbo@zaas.ac.cn (X.W.); zhouwd@zaas.ac.cn (W.Z.); 2 College of Biological and Environmental Science, Zhejiang Wanli University, Ningbo 315100, China; 2023881002@zwu.edu.cn (J.W.); 2024881073@zwu.edu.cn (Y.S.); 3 College of Information Engineering, China Jiliang University, Hangzhou 314423, China; xiangjun_li@cjlu.edu.cn

**Keywords:** biosensor, recombinase polymerase amplification, CRISPR/Cas12a, catalytic hairpin assembly, clinic diagnostic, the livestock and poultry environment

## Abstract

Duck plague virus (DPV), a highly contagious α-herpesvirus in the livestock and poultry environment, poses a significant threat to the healthy growth of ducks, potentially causing substantial economic losses. Effective control of DPV requires the development of specific diagnostic tools. A new fluorescent biosensor (R-C-CHA) was developed to detect virulent strains of DPV. It combined recombinase polymerase amplification (RPA), a CRISPR/Cas12a system, and catalytic hairpin assembly (CHA) for signal enhancement. The RPA primers were specifically designed to target the conserved DPV-CHv UL2 gene region, allowing for the rapid, efficient amplification of the target nucleic acids in isothermal conditions. The CRISPR/Cas12a system was used for sequence-specific recognition, activating its lateral cleavage activity. Furthermore, the CHA cascade reaction was utilized for enzyme-free fluorescent signal amplification. The results showed that the R-C-CHA biosensor completed the detection process in 40 min with a detection limit of 0.02 fg/μL, which was an approximate five-fold improvement compared to traditional RPA-CRISPR/Cas12a biosensors. The R-C-CHA biosensor also demonstrated perfect consistency with clinical detection and polymerase chain reaction (PCR) diagnosis, highlighting its strong potential for rapid detection in livestock and poultry farming settings.

## 1. Introduction

Duck plague virus (DPV), also known as duck enteritis virus, is a pathogen in the *Herpesviridae* family that primarily affects waterfowl, leading to tissue failure and hemorrhaging. Since its initial identification in the Netherlands, outbreaks of this infectious disease have been reported on duck farms worldwide [1,2,3]. Infected ducks exhibit symptoms such as systemic hemorrhage, intestinal lesions, and bleeding in the liver and spleen [4]. Given its ability to infect various duck breeds, the waterfowl breeding industry is at significant risk [5]. DPV outbreaks are highly lethal, with mortality rates reaching up to 90% in unvaccinated bird populations, causing substantial economic losses and trade restrictions in affected regions. Research shows that DPV initially replicates in the mucosa of the digestive tract before spreading to internal tissues, leading to necrotic bleeding in the intestines [6]. Although commercial vaccination plays a crucial role in disease prevention, a traditional attenuated live vaccine with UL2 gene-deficient is currently used for DPV. The antibodies induced by the vaccine cannot be distinguished from those caused by wild virus infection. Therefore, creating a fast, effective, highly sensitive method for on-site DPV virulent strain detection is essential for the early diagnosis and prevention of avian epidemics.

Various methods have been introduced for DPV identification, such as traditional virus isolation and culturing techniques, the enzyme-linked immunosorbent assay (ELISA), and the polymerase chain reaction (PCR) [7,8]. Traditional culturing methods combine morphological observations and microscopy in a controlled laboratory setting, which can take 2–3 d to yield definitive results, consequently limiting their accessibility in resource-constrained regions. Contrarily, although immunological methods such as ELISA can reduce the analysis time to a certain extent, they present challenges in pathogen detection due to their lower sensitivity, cross-reactivity with similar antigens, and varying antibody affinities toward target molecules. Most studies on pathogen identification rely on PCR, but its need for precise thermal cycle control limits its application in non-laboratory environments, especially in resource-poor areas [9]. Various nucleic acid amplification techniques have been employed to address these obstacles, including recombinase polymerase amplification (RPA) and loop-mediated isothermal amplification (LAMP), which improve the recognition sensitivity for pathogens in livestock and poultry. Liu et al. utilized the conserved Capripoxvirus (CapV) p32 gene region to develop RPA probes and primers. They also combined methods that involved real-time fluorescence RPA detection and naked-eye-visible lateral-flow band recognition for rapid, reliable CaPV identification [10].

The Clustered Regularly Interspaced Short Palindromic Repeats (CRISPR/Cas) system is a promising biosensing technology with promising applications in detection technology [11,12,13]. Since CRISPR/Cas systems, such as Cas14, Cas13, and Cas12, display enhanced programmability, sensitivity, simplicity, and specificity, they are often employed for pathogenic nucleic acid identification [14,15,16,17]. CRISPR/Cas12a stands out due to its trans-cleavage capability. This system involved binding between Cas12a and the stem loop structure of CRISPR RNA (crRNA), allowing it to recognize and cleave the dsDNA of the target near the protospacer adjacent motif [18]. A common approach for further improving the sensitivity of target DNA detection involves integrating CRISPR/Cas12a systems with various amplification strategies (e.g., LAMP and RPA) [19,20,21,22]. Zhou et al. utilized the precision of CRISPR/Cas12a and the efficiency of RPA technology to establish a novel avian influenza H5 virus detection method, which achieved a positive detection rate of 80.70% in 81 clinical samples [23]. Although these systems have potential, they still face issues in sensitivity, cost, and operability.

To address these problems, the CRISPR/Cas system integrates techniques to promote signal amplification and reduce background noise. The catalytic hairpin assembly (CHA), initially described by Yin et al. in 2008, is an effective enzyme-free, isothermal enhancement method [24,25,26]. It enhances the signal using entropy gain induced by thermodynamics. Various studies have shown that integrating CHA with diverse signaling mechanisms allows the rapid, highly sensitive, and specific detection of diverse targets, including proteins, nucleic acids, enzymes, metals, and cancer cells [27,28,29]. Yao et al. developed a technique for the identification of the influenza A virus by combining colloidal gold immunochromatography and CHA [30]. Song et al. employed the CRISPR/Cas13a system for CHA to develop a transient-wave fluorescence biosensing strategy. This allowed the amplification-free recognition of SARS-CoV-2 with a detection limit of 18.6 copies/µL, which was achieved within 50 min [31].

This work presents a novel fluorescence biosensor that integrates RPA, CRISPR/Cas12a, and CHA for fast, highly sensitive DPV-CHv identification. This approach leverages RPA for quick target amplification, CRISPR/Cas12a for precise sequence recognition, and CHA for exponential signal amplification, effectively addressing the sensitivity and specificity challenges of current rapid detection methods. By streamlining the testing process (from sample to result in 40 min) and validating it with clinical samples, the goal is to offer a transformative tool for controlling DPV transmission in high-risk settings, thereby safeguarding waterfowl production and global food security.

## 2. Methods and Materials

### 2.1. Regents and Instruments

All primers were synthesized at Tsingke Biotechnology (Nanjing, China). The information for all sequences is shown in Table 1 and Table S1. Ampure Future Biotechnology (Changzhou, China) provided the basic DNA rapid isothermal amplification kit, while the Lba Cas12a protein and FQ-ssDNA fluorescent reporter probe were purchased from Tolo Bio-Technology (Shanghai, China). The nucleic acid rapid lysis buffer was purchased from Kanglang Biotechnology (Shanghai, China), while the qPCR kit was obtained from BGI Tech Solutions (Nanjing, China). The fluorescent quantitative PCR instrument (Quant Studio 3) was acquired from Thermo Fisher Scientific Inc. (Waltham, MA, USA).

### 2.2. Virus Strain Sample Preparation

The chicken embryonated DPV-C-KCE attenuated strain (CVCC AV1222) and the standard DPV-CHv virulent strain (CVCC AV1221) were purchased from the China Veterinary Drug Administration. The goose parvovirus (GPV), influenza A virus (AIV-H9), reovirus (DRV), duck hepatitis virus (DHAV), and Tembusu virus (TMUV) strains were maintained by the Institute of Animal Husbandry and Veterinary Medicine at the Zhejiang Academy of Agricultural Sciences. Nucleic acid was automatically extracted from the purified virus strain sample and stored at −80 °C.

### 2.3. Primer, crRNA, and ssDNA Design and Synthesis

The DPV-CHv virulent strain (GenBank accession number: CVCC AV1221) was selected as the target, and the specific primers for its conserved sequences were designed. Three sets of RPA primers were created for the UL2 gene using Primer3Plus (https://www.primer3plus.com (accessed on 20 January 2025)). The specificity of the primers was verified against the GenBank database using BLAST(2.17.0) (https://blast.ncbi.nlm.nih.gov/Blast.cgi (accessed on 22 July 2025)). The Primer 5.0 software was employed to fabricate the specific primers and probes for PCR and qPCR. The corresponding crRNA was designed using the direct repeat sequence Dr region of Lba Cas12a, which targeted the amplification region adjacent to the prefix spacer sequence motif (PAM) site (5′-TTTN-3′) (See Supplementary Information Figure S1 for details). The hairpin structure required for the CHA reaction was also fabricated. Zixi Biotechnology (China, Beijing) synthesized all the primers and probes, which were diluted to 10 μmol/L with enzyme-free sterile water and stored at −20 °C.

### 2.4. Establishment of the R-C-CHA Detection Methods

A single reaction system was used to prepare the RPA-CRISPR/Cas12a, where Cas12a/crRNA-mediated cleavage and the RPA reaction occurred simultaneously. Table S2 shows the primary reagent components. The RPA-lyophilized precipitate was dissolved by adding the RPA primers and buffer to a PCR tube, which was mixed well via vortexing. Next, the Cas12a-crRNA complex was obtained by mixing the Cas12a enzyme, ssDNA reporter, and crRNA, followed by incubation at room temperature for 10 min. RPA reaction reagents and the CRISPR/Cas12a reaction components and RPA reagents were combined in the specified proportion, after which MgOAc and template nucleic acid were added, mixed rapidly, and centrifuged immediately.

H0 was used as the Cas12a trans-cleavage substrate and the CHA reaction initiator to combine the RPA-CRISPR/Cas12a system and CHA. A 10 mM Tris-HCl buffer consisting of 10 mM magnesium chloride and 500 mM sodium chloride (pH 7.5) was used to prepare the CHA nucleic acid solution. The hairpin structures were obtained by separately heating H1 and H2 to 95 °C for 5 min, followed by cooling to ambient temperature. The integrated experimental system contained Cas12a, crRNA, H0, H1/H2, and target gene DNA, while all reagents were dissolved in 1 × HOLMES buffer. All the reactions were carried out at 37 °C for 60 min using a real-time fluorescent PCR instrument. The reagent quantities are shown in Supplementary Information Tables S3 and S4.

### 2.5. Optimization of the R-C-CHA Biosensor

To maximize the analytical performance of the R-C-CHA biosensor, several crucial experimental RPA-CRISPR/Cas12-CHA parameters were optimized, including the Cas12a/crRNA ratio, the Cas12a concentrations, the hairpin concentration, and the H1/H2 ratio. To evaluate the performance of the R-C-CHA biosensor, a 2 nM DPV-CHv target DNA was used to measure the F/F_0_ value (signal-to-noise ratio). F and F_0_ denoted the R-C-CHA biosensor signal response with and without the target DNA sequence, respectively. The one-step RPA-CRISPR/Cas12a recognition method was employed to assess the optimal Cas12a protein and crRNA levels using Cas12a concentrations of 25 nM, 50 nM, 75 nM, 100 nM, 125 nM,150 nM, 175 nM, 200 nM, 225 nM, and 250 nM, respectively, and Cas12a/crRNA ratios of 4:1, 2:1, 1:1, 1:2, and 1:4. The CHA strategy in the reaction system was further optimized by adjusting several factors, such as the number of complementary bases on the hairpin, the hairpin concentration, the reaction temperature, the reaction time, the H1/H2 ratio, and the H1. We optimized the response of the hairpin induced on the stem with 8, 10, 12, and 14 complementary bases to the DPV-CHv DNA determination. In addition, the effects of 10, 20, 30, 40, 50, and 100 nM concentrations of hairpin-induced chains on the DPV-CHv DNA assay were also optimized. All experiments were performed in triplicate.

### 2.6. Specificity and Sensitivity Evaluation

Six common avian viruses were selected as interfering species to evaluate the specificity of the R-C-CHA biosensor. The process delineated in Section 2.2 was employed to extract the nucleic acids of these viruses. Sterile water was used as a blank control, while the other reaction components remained unchanged. After the reaction, the fluorescence values were determined via real-time quantitative PCR, and the fluorescence changes were observed visually to assess the specificity of the R-C-CHA biosensor.

To verify the sensitivity of the R-C-CHA biosensor, the procedure employed in Section 2.2 was used to obtain the DNA of the DPV virulent strain, which was diluted in a 10-fold gradient. A DNA concentration series of 10^−1^, 10^0^, 10, 10^2^, 10^3^, 10^4^, 10^5^, and 10^6^ fg/μL was tested for sensitivity using R-C-CHA and conventional RPA-CRISPR/Cas12a biosensors. To determine the lowest limit of detection (LOD) in the system, sterile water was used as a blank control, while the other reaction components remained unchanged. The reaction system followed the parameters listed in the table, and fluorescence values were read using a real-time fluorescence quantitative PCR instrument. The sensitivity of the R-C-CHA biosensor was evaluated by visually observing the fluorescence changes.

### 2.7. Analysis of Real Samples

Here, 10 Muscovy ducks (7 d old) were divided into two random groups, with 7 in the first and 3 in the second. The first group was challenged with 0.5 mL of the DPV-CHv pathogen via intramuscular injection (10^5^ TCID_50_) as a positive control, while the second group received 0.5 mL of sterile phosphate-buffered saline (PBS) injections as a negative control. All the ducks were housed in isolators and closely monitored continuously for clinical symptoms for 7 d. After treatment, throat swab samples were collected from both groups at 3 d, 4 d, 5 d, 6 d, and 7 d, respectively. Next, 1 μL of nucleic acid was extracted from the tissue samples using a rapid lysis solution as the target for R-C-CHA and qPCR detection, and the experimental results were recorded. The Ethics Committee of the Zhejiang Academy of Agricultural Sciences approved the sample collection process (Approval No.: 25ZALAS68).

### 2.8. Data Statistics and Analysis

All experiments were repeated in three parallels, and the data were expressed as mean ± standard deviation (SD). A Student’s *t*-test was employed for comparison between two samples, using the Graph Pad Prism software (version 8.0.2). Significance was denoted by values of *p* < 0.05 (*), *p* < 0.01 (**), *p* < 0.001 (***), and *p* < 0.0001 (****).

## 3. Results and Discussion

### 3.1. Principle of the R-C-CHA Biosensor

**Figure 1:**
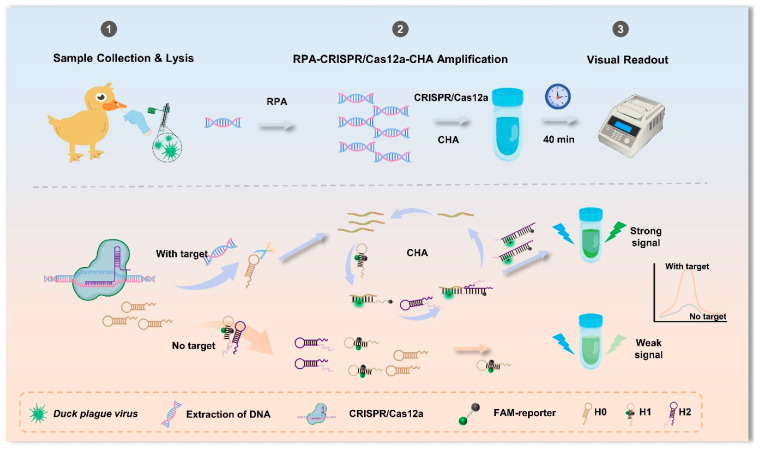
A schematic diagram of the fluorescence biosensor utilizing RPA-CRISPR/Cas12a technology for the detection of the DPV virulent strain via CHA.

This study developed a fluorescence biosensor based on RPA-CRISPR/Cas12a and CHA. As depicted in Figure 1, the collected duckbill swabs were subjected to extraction-free viral lysis for 2 min at 80 °C, followed by a one-step RPA-CRISPR/Cas12a reaction at 37 °C for 30 min. A hairpin reporter probe consisting of the thymine nucleotide specifically targeted by Cas12a and a primer chain that triggered the CHA reaction was designed [32]. When the target DNA was present, it was specifically captured by the CRISPR/Cas12a system crRNA. This initiated Cas12a trans-cleavage, which resulted in the cleavage of the H0 hairpin reporter probe H0 and the release of the primer strand. Adding the H1 and H2 hairpin probes to the reaction system triggered the subsequent CHA reaction, which was complete in 40 min without the need for any specialized equipment. Leveraging the high specificity and cleavage ability of CRISPR/Cas12a and the signal amplification capabilities of CHA, the R-C-CHA biosensor demonstrated excellent DPV-CHv detection performance.

### 3.2. Verification of the R-C-CHA Strategy

**Figure 2:**
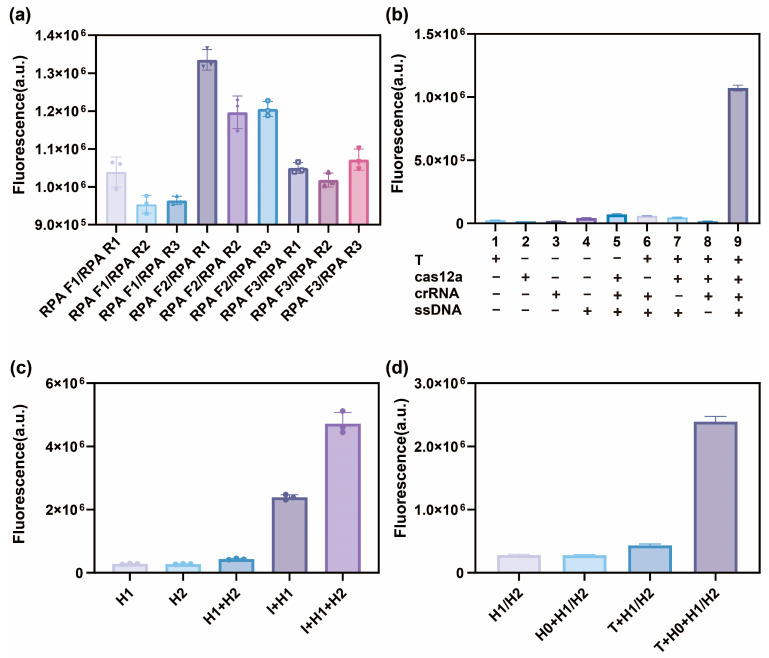
The feasibility of the fluorescence biosensor utilizing RPA-CRISPR/Cas12a technology for DPV virulent strain detection via CHA. (**a**) The fluorescence validation of the RPA primer screening. (**b**) The fluorescence response of the Cas12a test when exposed to various components. (**c**) The fluorescence response of the CHA test when exposed to various components. (**d**) The fluorescence response of the Cas12a-CHA test when exposed to various components.

The upstream and downstream primers of the RPA reaction design were divided into six groups: RPA F1/RPA R1, RPA F1/RPA R2, RPA F1/RPA R3, RPA F2/RPA R1, RPA F2/RPA R2, RPA F2/RPA R3, RPA F3/RPA R1, RPA F3/RPA R2, and RPA F3/RPA R3. These primers were purified for RPA and detected via fluorescence quantitative PCR. The fluorescence intensity values were used to determine the optimal primers. As shown in Figure 2a, the fourth set of primers (RPA F2/RPA R1) exhibited the highest detected fluorescence intensity values after amplification, which were selected for subsequent experiments.

Next, the feasibility and sensitivity of the one-step RPA-CRISPR/Cas12a approach for target DNA detection were verified. As shown in Figure 2b, a lower fluorescence signal was detected in the absence of any component in the Cas12a system, including the crRNA or target gene, since neither the cis nor the trans-cleavage activity of Cas12a was activated, which enhanced the ssDNA-FQ signal stability. The fluorescence signal rose rapidly in the presence of all the components, indicating that Cas12a/crRNA specifically recognized the target DNA and indiscriminately cleaved ssDNA with activated trans-cleavage activity.

Furthermore, the feasibility and sensitivity of the secondary signal amplification of the target DNA were verified after incorporation into the CHA system. As shown in Figure 2c, no fluorescent signal was evident since no labeled fluorescent dyes were added to H2, while H1 was present as a low fluorescent signal in the background. This was attributed to the close proximity of FAM and BHQ2 in the hairpin structure. Without initiator I, the H1/H2 fluorescent signal closely resembled that of H2, demonstrating the stable coexistence of these hairpin probes. The presence of initiator I substantially enhanced the fluorescent signal in the system, confirming that it triggered the CHA reaction. Figure 2d shows the fluorescent intensity values of the Cas12a-CHA system when exposed to different components. Without target DNA, the H1/H2 and H0 + H1/H2 reaction systems showed similar low fluorescent signals in the background. In the presence of target DNA and the absence of H0, the fluorescent intensity values of the R-C-CHA reaction system did not increase significantly. However, adding H0 increased the signal of the R-C-CHA reaction system, yielding higher fluorescent intensity values. Through the above series of control experiments, we can conclude that the reaction by-products that may be generated during the RPA and CRISPR/Cas12a processes will not cause CHA leakage. The experimental results demonstrate that the amplification reaction and the enhancement of fluorescence signal will only be initiated when all the R-C-CHA components are present.

### 3.3. Optimization of the R-C-CHA Biosensor Conditions

**Figure 3:**
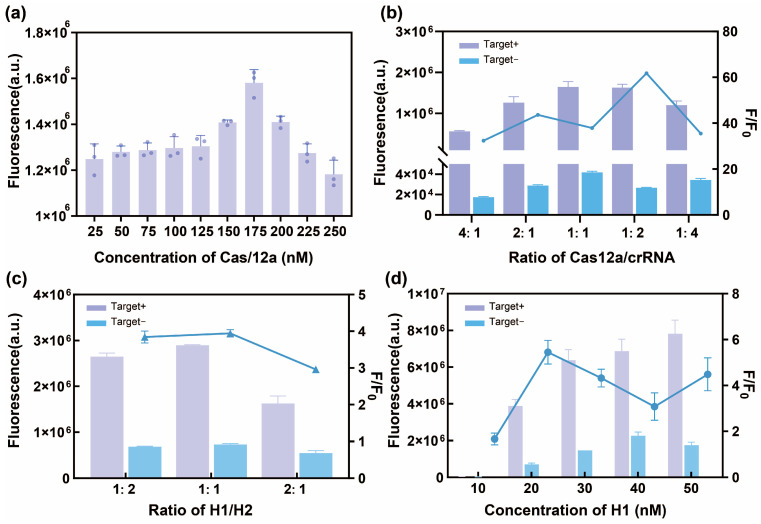
Optimization of the R-C-CHA biosensor conditions. (**a**) The Cas12a concentration. (**b**) The Cas12a/crRNA ratio. (**c**) The H1/H2 concentration ratio. (**d**) The H1 concentration. Each error bar represents one standard deviation, calculated from at least three measurements. The lines in the figure represent the trend of F/F_0_ value under different optimization conditions.

Achieving the best performance from the R-C-CHA biosensor required the optimization of several critical parameters, such as the Cas12a/crRNA ratio and the reporter gene concentration, as well as the incubation temperature and time. To minimize the impact of background signals on experimental results, we used the ratio of target containing group to non-target group results (F/F_0_) as the basis for selecting the optimal conditions that yield the strongest signal with the lowest background. The Cas12a concentration was optimized first. As shown in Figure 3a, the fluorescence intensity reached maximum levels at Cas 12a concentrations of 175 nM. Therefore, 175 nM was selected as the optimal Cas12a concentration for the CRISPR/Cas12a system. Next, the optimal Cas12a/crRNA ratio was determined. As shown in Figure 3b, the F/F_0_ value peaked at a 1:2 Cas12a/crRNA ratio. The CHA strategy was employed to further optimize the reaction system, including the H1/H2 ratio, the H1 concentration, the number of complementary bases induced by the stem-loop structure, the hairpin structure concentration, and the reaction temperature and time. As shown in Figure 3c,d, the F/F_0_ value was highest at an H1/H2 ratio of 1:1 and an H1 concentration of 20. Details regarding the other optimization conditions of the R-C-CHA biosensor can be found in the Supporting Information Figure S2.

### 3.4. Analytical Performance of the R-C-CHA Biosensor

**Figure 4:**
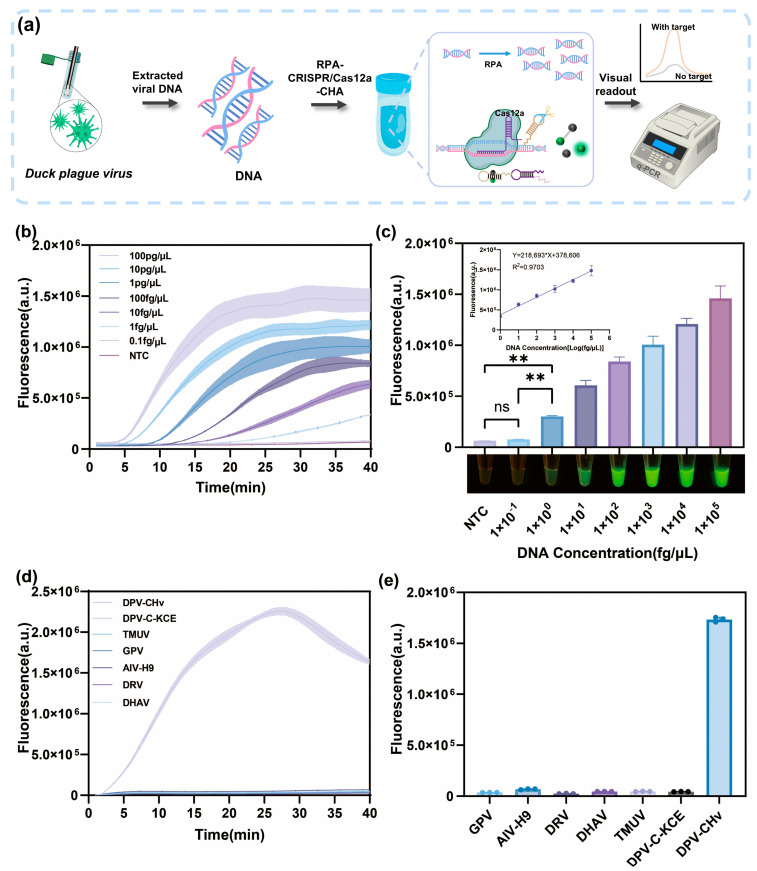
(**a**) The principle of the R-C-CHA biosensor that integrates RPA, CRISPR/Cas12a, and CHA for DPV-CHv detection. (**b**) The typical fluorescence curves of the R-C-CHA biosensor for various DPV-CHv concentrations. (**c**) The fluorescence intensity of the R-C-CHA biosensor for the DPV-CHv DNA. The inset shows the linear equation for this approach in a concentration range of 1 × 10^−1^ fg/μL to 1 × 10^5^ fg/μL. The images showing color variations denote the concentrations ranging between 1 × 10^−1^ fg/μL and 1 × 10^5^ fg/μL. (**d**) The typical fluorescence curves of the R-C-CHA biosensor for using common waterfowl viruses, including MDPV, GPV, AIV-H9, DRV, DHAV, TMUV, and DPV-C-KCE DNA sequences. (**e**) The fluorescence intensity of the R-C-CHA biosensor for the different waterfowl viruses (** represents *p* < 0.01; ns represents no significant). The error bars were produced via triplicate experiments.

To demonstrate the sensitivity of the R-C-CHA biosensor for DPV detection in the optimized conditions, a series of samples containing DPV-CHv was prepared. Figure 4a shows the steps for DPV-CHv DNA identification. A calibration curve was generated at DPV-CHv DNA concentrations of 1 × 10^−1^, 10^0^, 10^1^, 10^2^, 10^3^, 10^4^, and 10^5^ fg/μL (Figure 4b,c) to illustrate the association between the fluorescence intensity and the logarithmic DPV-CHv level. A strong linear correlation was observed, with a regression equation of Y = 218,693 + 378,606X and a correlation coefficient (R^2^) of 0.9703. Here, Y represented the fluorescence intensity at 520 nm, and X represented the logarithmic DPV-CHv concentration in fg/μL. The LOD was calculated as 0.02 fg/μL using the formula LOD = 3σ/K, where σ represented the standard deviation of the blank sample and K denoted the standard curve slope.

The recognition sensitivity of the R-C-CHA biosensor was approximately five-fold higher than that of RPA-CRISPR/Cas12a. Supplementary Information Figure S3 shows the RPA-CRISPR/Cas12a biosensor detection results. In the R-C-CHA biosensor, the integration of CHA can significantly suppress background fluorescence even in the absence of target DNA, thereby increasing the ratio of signal to noise. Furthermore, the R-C-CHA biosensor was compared with qPCR, considered the optimal method for detecting DPV due to its high sensitivity and accuracy. The findings indicated showed that the R-C-CHA biosensor and qPCR showed comparable sensitivity (Figure S4).

Furthermore, this study assessed the specificity of the R-C-CHA biosensor for DPV-CHv recognition using common waterfowl viruses, including GPV, AIV-H9, DRV, DHAV, TMUV and DPV-C-KCE. As illustrated in Figure 4d,e, these viruses did not significantly change the fluorescence values compared to the experimental group, indicating that the constructed R-C-CHA biosensor exhibited high specificity for DPV-CHv.

### 3.5. Clinical Sample Testing and Evaluation

**Figure 5:**
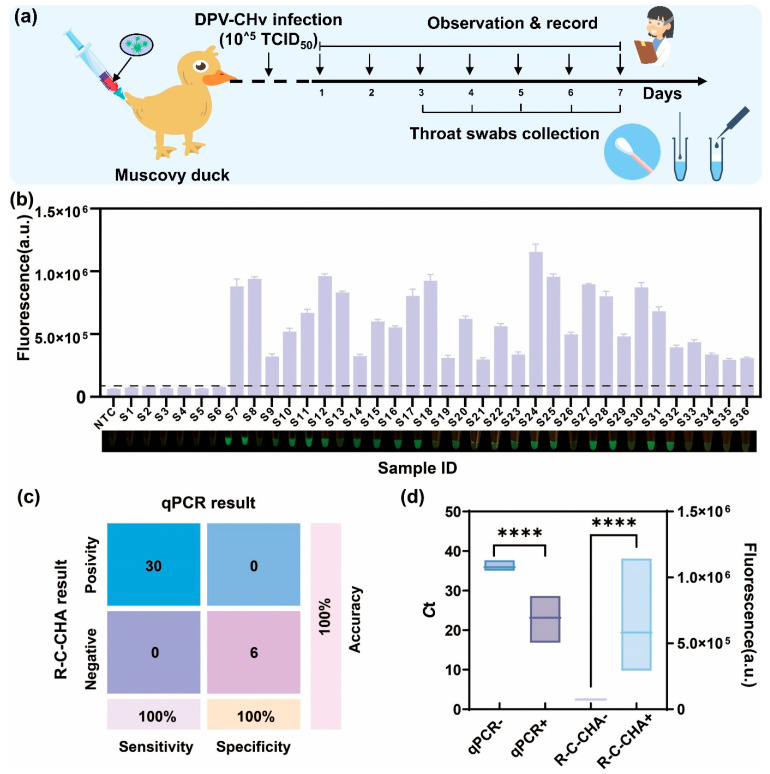
The detection of DPV-CHv in clinical samples using the R-C-CHA biosensor. (**a**) A schematic diagram of DPV-CHv infection in Muscovy ducks. (**b**) A comparison between the R-C-CHA biosensor and qPCR results for 36 actual samples. The endpoint R-C-CHA biosensor results are depicted as bar charts. The error bars represent the means ± standard deviation from three technical repetitions. NTC: non-template control. The horizontal dashed line indicates the threshold fluorescence intensity. The fluorescent R-C-CHA biosensor images are also included. (**c**) The confusion matrix illustrating the comparison between the performance characteristics in the qPCR and R-C-CHA biosensor results. (**d**) The box plots comparing the qPCR and R-C-CHA biosensor results of the positive and negative samples (**** represents *p* < 0.0001). The error bars denote means ± standard deviation after three technical repetitions.

The rapid, accurate, and sensitive detection of DPV-CHv is the most effective way to stop disease propagation. To verify the clinical DPV-CHv recognition performance, a certain quantity of DPV-CHv was injected into Muscovy ducks, after which oral swab samples were collected at different time points (Figure 5a). Then, 36 deidentified clinical throat swab samples were obtained, after which a commercial nucleic acid extraction kit was employed to acquire the DNA. Next, qPCR was employed to confirm the presence of 30 positive and six negative DPV-CHv samples before using the R-C-CHA biosensor assay to test the DNA specimens. Figure 5b shows the fluorescence intensity values after exposure to the R-C-CHA biosensor. The negative control samples were employed for background subtraction to determine the fluorescence threshold, which was set as the negative sample median (mean + 3σ), represented by a black dotted line (Figure 5b). The same clinical samples were subjected to qPCR analysis, and the resulting Ct values effectively differentiated positive from negative samples (Table S6), showing consistency with the R-C-CHA biosensor readings. To facilitate direct comparison, a correlation analysis was conducted between the qPCR Ct values and the fluorescence signals obtained from the R-C-CHA biosensor (Figure S5), revealing a statistically significant correlation. This concordance between the two analytical methods underscores the accuracy and reliability of the RPA-CRISPR/Cas12a-CHA biosensor for the detection of DPV in real clinical specimens. According to the confusion matrix in Figure 5c, this detection method displayed 100% sensitivity, specificity, and overall accuracy in recognizing the highly virulent DPV strain. This highlighted its ability to effectively distinguish between true positive and negative results in clinical duck samples. The box plot in Figure 5d compares the detection results of the two methods in the positive (*n* = 30) and negative samples (*n* = 6). In qPCR results, A Ct value of 32 or higher in the qPCR results was considered negative. The qPCR- and qPCR+ groups denoted the negative and positive samples, respectively, with Ct values of approximately 35 and 20. Furthermore, the results indicated that the fluorescence intensity of the R-C-CHA group was significantly higher than in the R-C-CHA+ group. This demonstrated that the sensitivity of the R-C-CHA biosensor was superior to the qPCR method for DPV-CHv detection.

## 4. Conclusions

Duck plague virus (DPV) is a highly contagious pathogen that causes severe infectious disease in ducks, leading to substantial economic losses in the waterfowl industry [33]. Therefore, the development of rapid and accurate diagnostic methods is critical for effective disease prevention and control. Conventional PCR-based assays are widely used for DPV detection due to their high specificity and reliability. However, their analytical sensitivity is typically limited to approximately 1 pg/μL^−1^, and they require thermal cycling and laboratory-based instrumentation, which restricts their applicability in field or point-of-care settings [34]. LAMP offers the advantage of isothermal operation and reduced equipment requirements, but its detection limit for DPV remains at a similar level (~1 pg/μL) and the complex primer design may increase the risk of nonspecific amplification [35]. RPA represents a significant advancement in isothermal nucleic acid amplification, achieving a markedly improved detection limit of approximately 1 fg/μL within less than one hour [36]. Nevertheless, RPA-based assays alone rely primarily on bulk amplification signals, which may be susceptible to background interference when detecting ultra-low target concentrations (See Supplementary Information Table S5).

To further position the proposed method within the broader context of CRISPR-based diagnostic technologies, a comparative analysis with other established CRISPR-assisted amplification platforms was conducted, as summarized in Table 2. Previously reported platforms, such as LAMP–CRISPR [37], RAA–CRISPR [38,39], and RPA–CRISPR [40], have demonstrated promising performance for the detection of various pathogens, with limits of detection ranging from 100 fg/μL to 1 fg/μL and assay times typically exceeding 40 min. In comparison, the RPA–CRISPR/Cas12a–CHA biosensor developed in this work achieved a notably lower detection limit of 0.02 fg/μL within 40 min, without increasing assay duration. Overall, this comparative evaluation indicates that the introduction of CHA provides a clear sensitivity advantage over conventional CRISPR-based amplification strategies, while preserving the simplicity and rapid response characteristics of isothermal CRISPR diagnostics.

This study used RPA-CRISPR/Cas12a-CHA technology to construct a novel R-C-CHA biosensor for highly sensitive DPV-CHv identification, which integrates isothermal amplification, CRISPR/Cas12a, and enzyme-free cascade signal amplification. The proposed biosensor showed sensitivity at a concentration of 0.02 fg/μL level in the 10^−1^~10^5^ fg/μL range and exhibited excellent specificity and stability in practical applications. Compared with conventional RPA–CRISPR/Cas12a assays and other reported CRISPR-based amplification platforms, the introduction of CHA enabled a notable enhancement in signal output and sensitivity without increasing assay time, highlighting the advantage of the multi-level amplification strategy. Importantly, this enzyme-free cascade amplification enables the use of lower concentrations of Cas12a enzyme and fluorescent reporters compared with conventional RPA-CRISPR/Cas12a assays, effectively reducing reagent consumption and overall assay cost. Moreover, the R-C-CHA biosensor demonstrated sensitivity comparable to that of qPCR while maintaining isothermal operation and simplified instrumentation requirements, underscoring its potential as an alternative molecular diagnostic tool for DPV detection. The R-C-CHA biosensor offers exceptional versatility and can be used to identify a broad range of pathogenic microorganisms by modifying the target recognition sequence. Future research directions include combining the R-C-CHA biosensor with microfluidic systems to simplify the procedures and reduce environmental pollution, as well as the developing portable fluorescence detection equipment for POCT detection.

## Figures and Tables

**Table 1:** The nucleic acid sequences used in this study.

Amplification Method	Primer	Primers Sequence (5′-3′)
RPA	RPA F1	GCTTTGGCCCATGCCTCTAGGCAGCCATGATC
	RPA F2	CGAACGGCCGATAATATATTACGTAGGCTAG
	RPA F3	TATATTACGTAGGCTAGGAGGTATCTGAATAC
	RPA R1	CATGGACGAGGTACTGTGCTCCATCGGATG
	RPA R2	GAACGGCGCTGTGACATCGAAGAAGTCCTGC
	RPA R3	CTCGAGTATTACTTGAGTATGAACGGCGCTGTG
	RPA P	CTGAATGCGAGCCCGTGAGCCTGGCCGGGT (dT-FAM) G (dSpacer) (dT-BHQ1) GATATGGATCTTGCC
CRISPR	CrRNA	AAUUUCUACUAAGUGUAGAUCCCAACUAUGAUGACUUUUA
	hairpin reporter8	CCGTAAGTTTTTTCCTACTCTCAACTAACTTACGG
	hairpin reporter10	CCGTAAGTTATTTTTCCTACTCTCAACTAACTTACGG
	hairpin reporter12	CCGTAAGTTAGTTTTTTCCTACTCTCAACTAACTTACGG
	hairpin reporter14	CCGTAAGTTAGTTGTTTTTCCTACTCTCAACTAACTTACGG
CHA	I	CCTACTCTCAACTAACTTACGG
	H1	[FAM]-CCGTAAGTTAGTTGAGAGTAGGGGAGACCATGTCCTACTCTCAACTAAC-[BHQ1]
	H2	GAGAGTAGGACATGGTCTCCCCTACTCTCAACTAACGGAGACCATGT

**Table 2:** Comparison of our developed method with the other established CRISPR-based amplification platforms.

Assay	The Object of Detection	Limit of Detection	Time	References
LAMP-CRISPR	Nocardia farcinica	100 fg/μL	70 min	[37]
RAA-CRISPR	Cyprinid Herpesvirus-3	100 ag/μL	1 h 40 min	[38]
RAA-CRISPR	Cladobotryum mycophilum	2 fg/μL	40 min	[39]
RPA-CRISPR	Heterodera schachtii	1 fg/μL	<1 h	[40]
RPA-CRISPR-CHA	DPV	0.02 fg/μL	40 min	This work

## Data Availability

The data analyzed during the study are available from the corresponding author upon reasonable request.

## Supplementary Materials

The following supporting information can be downloaded at: https://www.mdpi.com/article/10.3390/bios16020073/s1, Figure S1: Oligo design for duck plague virus (DPV) virulent strain; Figure S2: Optimization results of R-C-CHA biosensor; Figure S3: The fluorescence intensity of the RPA-CRISPR/Cas12a biosensor for the DPV-CHv DNA; Figure S4: Sensitivity test results of DPV-CHv fluorescence quantitative PCR; Figure S5: Correlation analysis between Ct values from clinical samples detection via qPCR and fluorescence signals from R-C-CHA Biosensors; Table S1: Nucleic acid sequences for PCR used in this work; Table S2: One-Pot RPA/CRISPR Detection System; Table S3: CHA Detection System; Table S4: R-C-C Detection System; Table S5: Comparison of our developed method with the existing DPV detection techniques; Table S6: Clinical sample test results: qPCR detection cycle threshold (Ct value) and fluorescence intensity value of the R-C-CHA biosensor.
