# Supplementary material for: A RPA-CRISPR/Cas12a-Powered Catalytic Hairpin Assembly Fluorescence Biosensor for Duck Plague Virus Virulent Strain Detection
Source: Biosensors (Basel). 2026 Jan 26;16(2):73. doi: 10.3390/bios16020073 (PMC12938105; doi:10.3390/bios16020073)
Supplement: Supplementary file 1 [file biosensors-16-00073-s001.zip › biosensors-4107146-supplementary.pdf]

Supplementary Information

A RPA-CRISPR/Cas12a-Powered Catalytic Hairpin Assembly Fluorescence Biosensor for Duck Plague Virus Virulent Strain Detection

Yue Wu [1,†], JiaxinWan [2,†], Xingbo Wang [1], Yunjie Shen [2], Xiangjun Li [3], Weidong Zhou [1], Yinchu Zhu [1,*] and Xing Xu [1,*]

[1] State Key Laboratory for Quality and Safety of Agro-Products, Institute of Animal Husbandry and Veterinary Science, Zhejiang Academy of Agricultural Sciences, Hangzhou 310021, China; wuyue@zaas.ac.cn (Y.W.); wangxingnbo@zaas.ac.cn (X.W.); zhouwd@zaas.ac.cn (W.Z.)

[2] College of Biological and Environmental Science, Zhejiang Wanli University, Ningbo 315100, China; 2023881002@zwu.edu.cn (J.W.); 2024881073@zwu.edu.cn (Y.S.)

[3] College of Information Engineering, China Jiliang University, Hangzhou 314423, China; xiangjun_li@cjlu.edu.cn

* Correspondence: zhuyinchu@zaas.ac.cn (Y.Z.); xvxing@zaas.ac.cn (X.X.)

† These authors contributed equally to this work.

Based on all DPV sequences in GenBank, gene sequence alignment revealed that the UL2 gene in the attenuated duck plague strain lacks a 528 bp B fragment compared to the virulent strain (the deleted segment spans nucleotides 198–725 in the virulent strain; see Figure S1). The primers for this experiment were designed based on the B fragment.

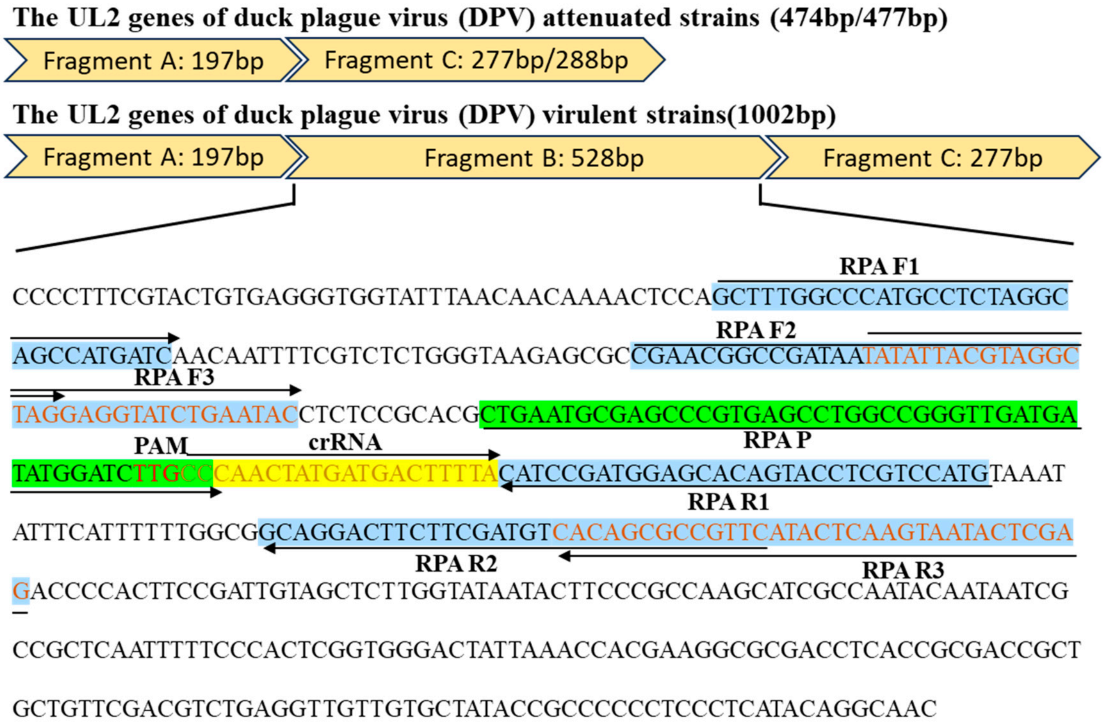

Figure S1. Oligo design for the virulent strain of the duck plague virus (DPV). RPA primers are highlighted in blue. The RPA probe is highlighted in green. The design of crRNA based on PAM sites in RPA amplification sequences is highlighted in yellow, while the PAM sites are indicated in red.

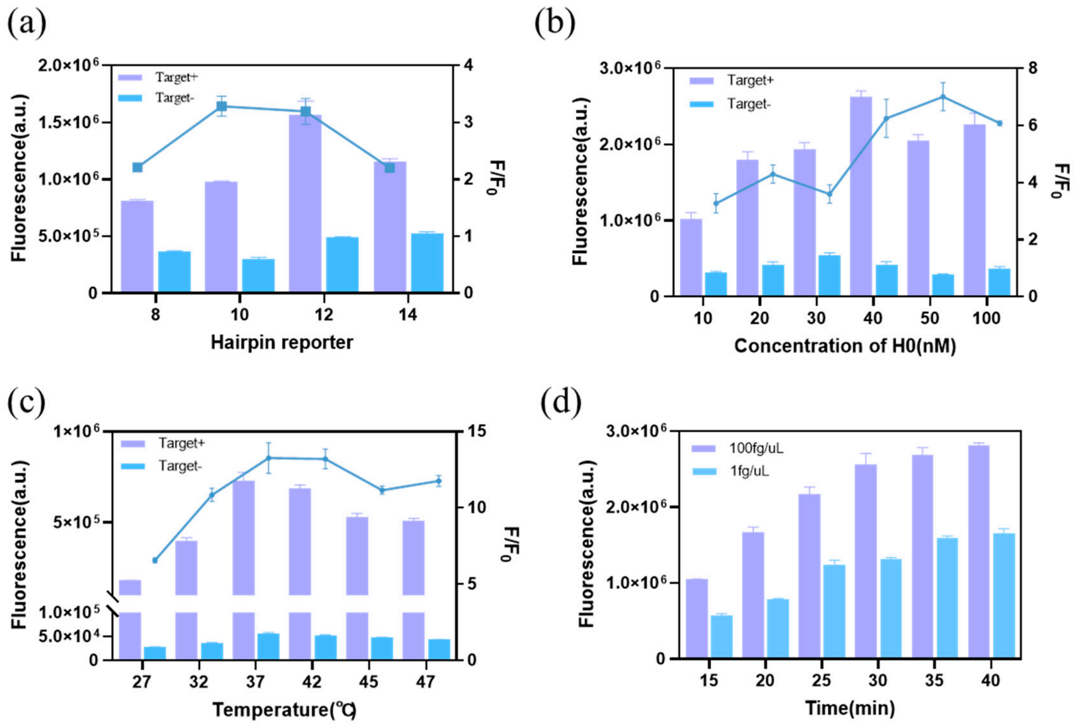

Figure S2. Optimization results of R-C-CHA biosensor. (a) Optimization of hairpin reporter probe in the R-C-CHA system; F and F_0 is the fluorescence intensity of the R-C-CHA system with and without target DNA, and the concentration of target DNA is 1 nM. (b) Optimization of H0 concentration. (c) Optimization of reaction temperature. (d) Optimization of reaction time.

A strong linear correlation was observed, with a regression equation of Y=211189 + 100520X and a correlation coefficient (R^2) of 0.9784. Here, Y represented the fluorescence intensity at 520 nm, and X represented the logarithmic DPV-CHv concentration in fg/μL. The LOD was calculated as 0.1 fg/μL using the formula LOD=$3\sigma/k$, where σ represented the standard deviation of the blank sample and k denoted the standard curve slope.

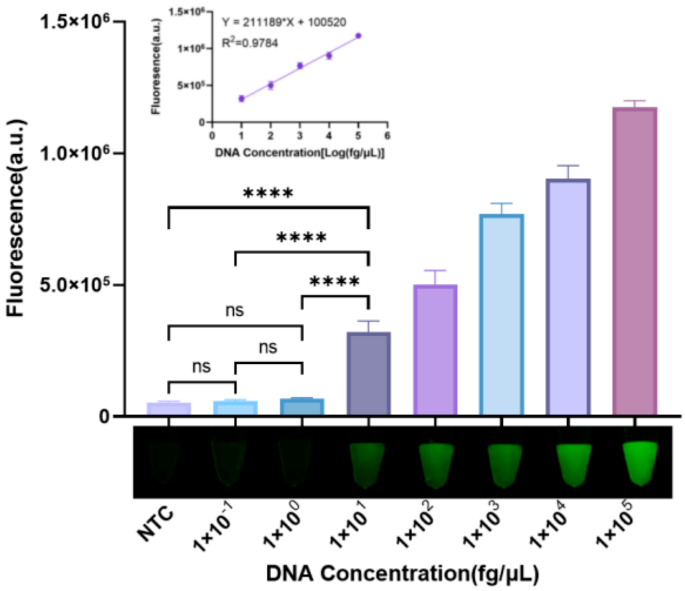

Figure S3. The fluorescence intensity of the RPA-CRISPR/Cas12a biosensor for the DPV-CHv DNA. The inset shows the linear equation for this approach in a concentration range of 1×10^{-1} fg/μL to 1×10^{5} fg/μL. The images showing color variations denote the concentrations ranging between 1×10^{-1} fg/μL and 1×10^{5} fg/μL.

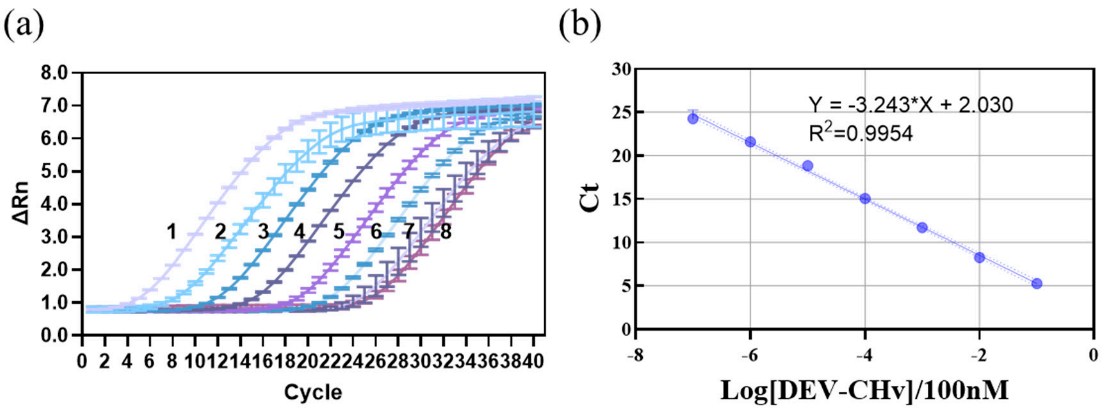

Figure S4. Sensitivity test results of DPV-CHv fluorescence quantitative PCR. (a) Fluorescence quantitative PCR curve. The standard samples were diluted by 10^1 to 10^8 times, respectively, and the reaction curves after amplification using these diluted samples as templates were obtained. (b) The PCR detection showed linearity within six orders of magnitude, and the detection sensitivity was 2×10^{-7} nM. Each reaction was performed with 3 replicates.

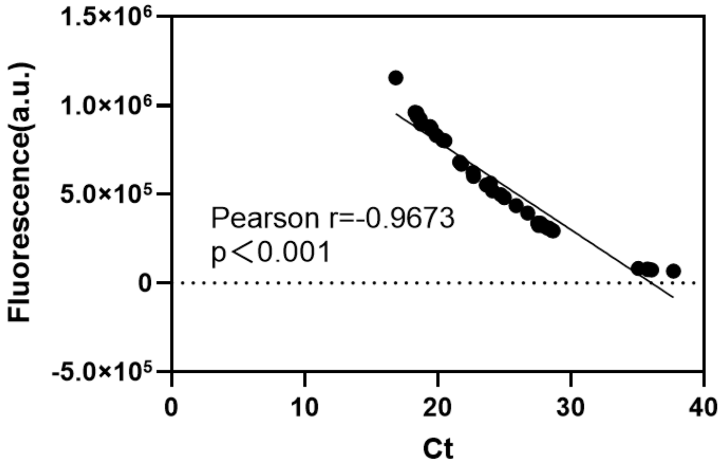

Figure S5. Correlation analysis between Ct values from clinical sample detection via qPCR and fluorescence signals from R-C-CHA biosensors.

Table S1. Nucleic acid sequences for PCR used in this work.

Amplification method	Primer name	Sequence （5′-3′）
PCR	PCR-F	TGACCAACCAACGTCTACATGC
	PCR-R	CAGAAAGCCTTAAATTCAGCGTG
qPCR	qPCR-F	TGGGCAAGATCCATATCATCAA
	qPCR-R	CCTCTCCGCACGCTGAAT
	qPCR-P	FAM-CGGCCAGGCTCACGGGCTC-BHQ1

Table S2. One-Pot RPA/CRISPR Detection System.

Reagent	Component	Volume	Final concentration
A: RPA reagents	A buffer	29.5 μL	/
	forward primer (10 μM)	1~2.5 μL	200~500 nM
	reverse primer (10 μM)	1~2.5 μL	200~500 nM
	Nuclease-Free Water	supplemented to 48μL	/
	total	48 μL was added to the dry powder pellet (Amp-Future); 8 μL was taken for a single 20 μL RPA/CRISPR system for the assay experiment.	
B: Cas12a reagents	Cas12a buffer (10×)	2 μL	1×
	ssDNA reporter (10 μM)	0.4~1 μL	200~500 nM
	LbCas12a (5 μM)	0.1~1 μL	25~250 nM
	crRNA (5 μM)	0.1~1 μL	25~250 nM
	Nuclease-Free water	supplemented to 5 μL	/
MgOAc (280 nM)		1 μL was added before the reaction.	14 nM
RNA/DNA template		0~9 μL	
Nuclease-Free water		Adjust the total reaction volume to 20 μL according to the volume of the nucleic acid template.	

Table S3. CHA Detection System.

Reagent	Component	Volume	Final concentration
CHA	I (1 μM)	0.2~1 μL	10~50 nM
	H1 (10 μM)	0.5~1 μL	250~500 nM
	H2 (10 μM)	0.5~1 μL	200~500 nM
	Tris-HCl buffe	supplemented to 20μL	/
Reaction		37℃，10min	

Table S4. R-C-C Detection System.

Reagent	Component	Volume	Final concentration
A: RPA reagents	A buffer	29.5 µL	/
	forward primer (40 µM)	1~2.5 µL	200~500 nM
	reverse primer (40 µM)	1~2.5 µL	200~500 nM
	Nuclease-Free Water	supplemented to 48µL	/
	total	48 µL was added to the dry powder pellet (AmpFuture), 8 µL was taken for a single 20 µL RPA/CRISPR system for the assay experiment.	
B: Cas12a reagents	Cas12a buffer (10×)	2 µL	1×
	ssDNA reporter (10 µM)	0.4~1 µL	200~500 nM
	LbCas12a (5 µM)	0.1~1 µL	25~250 nM
	crRNA (5 µM)	0.1~1 µL	25~250 nM
	Nuclease-Free water	supplemented to 5 µL	/
MgOAc (280 nM)		1 µL, was added before the reaction.	14 nM
RNA/DNA template		0~9 µL	
Nuclease-Free water		Adjust the total reaction volume to 20 µL according to the volume of the nucleic acid template.	
Reaction		37℃，30min	
CHA	H0 (1 µM)	0.2~2 µL	10~100 nM
	H1 (10 µM)	0.5~1 µL	250~500 nM
	H2 (10 µM)	0.5~1 µL	200~500 nM
	Tris-HCl buffe	supplemented to 20µL	/
Reaction		37℃，10min	

Table S5: Comparison of our developed method with the existing DPV detection techniques.

Assay	Limit of detection	Time	References
PCR	1pg/µl	30min	[1]
LAMP	1pg/µl	30min	[2]
RPA	1 fg/µl	<1h	[3]
RPA-CRISPR/Cas12	0.7fg/µl	40 min	This work
RPA-CRISPR/Cas12-CHA	0.02fg/µl	40min	This work

Table S6. Clinical sample test results: qPCR detection cycle threshold (Ct value) and fluorescence intensity value of the R-C-CHA biosensor.

Sample	1	2	3	4	5	6	7	8	9	10	11	12	13	14	15	16	17	18
qPCR(Ct)	36.05	35.06	37.69	35.78	37.68	35.75	19.41	18.44	27.92	24.09	21.76	18.27	19.87	27.55	22.67	23.64	20.35	18.66
	-	-	-	-	-	-	+	+	+	+	+	+	+	+	+	+	+	+
R-C-CHA (F)	-	-	-	-	-	-	+	+	+	+	+	+	+	+	+	+	+	+
	73741.35	83000.86	67151.30	75300.28	65860.86	78365.36	880233.98	939788.66	321752.66	519139.31	669487.74	961321.32	830658.53	324438.87	600982.60	552912.21	804108.81	924892.18

Sample	19	20	21	22	23	24	25	26	27	28	29	30	31	32	33	34	35	36
qPCR(Ct)	28.21	22.64	28.54	23.93	27.70	16.83	18.42	24.73	18.72	20.53	24.97	19.49	21.64	26.75	25.88	27.50	28.65	28.35
	+	+	+	+	+	+	+	+	+	+	+	+	+	+	+	+	+	+
R-C-CHA (F)	+	+	+	+	+	+	+	+	-	+	-	+	+	+	+	+	+	+
	310312.84	621814.06	297190.37	563035.25	337007.51	1155339.41	956679.68	497293.43	896472.02	800397.96	480979.70	871619.83	681369.24	394385.36	435254.03	337104.15	294037.80	309495.88

Reference

[1] Liji X., Huang L., et al. Development of a polymerase chain reaction assay for differentiation of duck plague virus virulent strains from attenuated strains. Progress in Veterinary Medicine, 38(2017).

[2] Grzegorz W, Elzbieta S.S. First survey of the occurrence of duck enteritis virus (DEV) in free-ranging Polish water birds. Archives of Virology, 12(2013).

[3] Jiaxin W, Yinchu Z, Xing X, et al. Development of a rapid detection method for a virulent strain of duck enteritis virus based on real-time fluorescence recombinase polymerase amplification. Chinese Journal of Biotechnology, 2025.
